# Exploring perceptions of community health policy in Kenya and identifying implications for policy change

**DOI:** 10.1093/heapol/czv007

**Published:** 2015-03-26

**Authors:** Rosalind McCollum, Lilian Otiso, Maryline Mireku, Sally Theobald, Korrie de Koning, Salim Hussein, Miriam Taegtmeyer

**Affiliations:** ^1^Department of International Public Health, Liverpool School of Tropical Medicine, Pembroke Place, Liverpool, UK,; ^2^Research and Strategic Information Department LVCT Health, Nairobi, Kenya,; ^3^KIT Health, Royal Tropical Institute, Amsterdam, The Netherlands, and; ^4^Community Health and Development Unit, Ministry of Health, Kenya

**Keywords:** Community health extension worker, community health worker, close to community provider, Kenya, policy

## Abstract

**Background:** Global interest and investment in close-to-community health services is increasing. Kenya is currently revising its community health strategy (CHS) alongside political devolution, which will result in revisioning of responsibility for local services. This article aims to explore drivers of policy change from key informant perspectives and to study perceptions of current community health services from community and sub-county levels, including perceptions of what is and what is not working well. It highlights implications for managing policy change.

**Methods:** We conducted 40 in-depth interviews and 10 focus group discussions with a range of participants to capture plural perspectives, including those who will influence or be influenced by CHS policy change in Kenya (policymakers, sub-county health management teams, facility managers, community health extension worker (CHEW), community health workers (CHWs), clients and community members) in two purposively selected counties: Nairobi and Kitui. Qualitative data were digitally recorded, transcribed, translated and coded before framework analysis.

**Results:** There is widespread community appreciation for the existing strategy. High attrition, lack of accountability for voluntary CHWs and lack of funds to pay CHW salaries, combined with high CHEW workload were seen as main drivers for strategy change. Areas for change identified include: lack of clear supervisory structure including provision of adequate travel resources, current uneven coverage and equity of community health services, limited community knowledge about the strategy revision and demand for home-based HIV testing and counselling.

**Conclusion:** This in-depth analysis which captures multiple perspectives results in robust recommendations for strategy revision informed by the Five Wonders of Change Framework. These recommendations point towards a more people-centred health system for improved equity and effectiveness and indicate priority areas for action if success of policy change through the roll-out of the revised strategy is to be realized.

**Key Messages**

- There was widespread appreciation of the CHS, with positive evaluation of CHWs in particular.
- Sustainability, funding, workload and accountability challenges were identified as the main drivers for change of the CHS.
- The sustainability and feasibility of the CHS revision will depend on commitment across all levels. Community engagement, management of provider expectations and decision makers' support at county level will be key to success.

## Introduction

Kenya's interest and investment in close-to-community (CTC) health services is growing, with substantial commitment from the Government of Kenya, Non-Governmental Organizations (NGOs) and donors (UNICEF 2010; Ministry of Medical Services and Ministry of Public Health and Sanitation 2012). This mirrors a renewed global interest in this approach. (Global Health Workforce Alliance 2012; Naimoli *et al*. 2012; The Earth Institute Columbia University 2013). CTC providers^1^ can play an important role in increasing access to care and services, through their unique position as embedded community members who can forge a link between their community and the formal health system, taking into account social and environmental determinants for health (Bhutta *et al*. 2010).

Policymakers and governments making decisions about CTC programmes need to balance equity and effectiveness targets with planning for financing, training, workload, supervision and motivation of CTC providers, community engagement and evidence-based decision making which utilizes programme data. Given the evidence for the effectiveness of using CTC providers to reduce maternal and child morbidity and mortality (Bhutta *et al*. 2010; Lewin *et al*. 2010), policymakers need to interpret these findings in light of variations in context, programme design and quality (Glenton *et al*. 2013).

Performance of CTC providers can be inconsistent. CTC providers working with vertical disease-focused programmes, such as tuberculosis and HIV, with tailored supervision structures often perform better than those with a more integrated long-term approach (Alamo *et al*. 2012). This tendency for vertical-programme-focused policy has the potential to generate competition for resources and poor coordination among stakeholders and may ultimately undermine policymakers’ targets to realize universal health coverage (Victora *et al*. 2004).

### Kenya community health strategy

Health service provision in Kenya is centred around four tiers of service provision—community, primary care, primary (county) referral and tertiary (national) referral services (Ministry of Medical Services and Ministry of Public Health and Sanitation 2012). Under the new decentralized strategy, sub-counties are responsible to deliver health services and implement health programmes (National Coordinating Agency for Population and Development *et al*. 2010).

**Figure 1:**
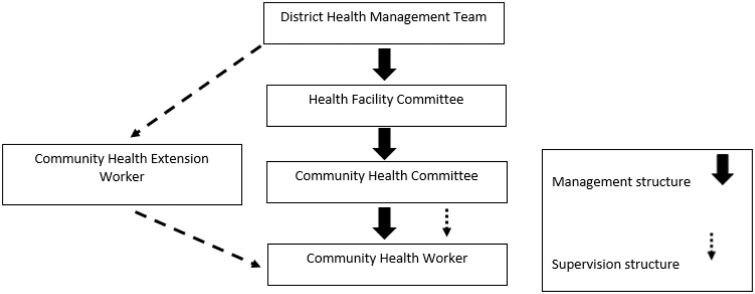
Organogram showing CHS management and supervision structure

The Kenya community health strategy (CHS), rolled out in 2006 (Ministry of Health 2006), provides a plan to expand community access to health care across all stages of the life cycle. Within this strategy the district health management team (DHMT), now called sub-county health management team (SCHMT), is responsible for the coordination of community services, with a focal person tasked with the supervision, planning and monitoring of community health-related activities. Community health services within the sub-county are centred around community units (described as ‘level 1 units’). Each such unit consists of 5000 people including 50 volunteer community health workers (CHWs) responsible for 20 households each. The strategy lays out their roles and responsibilities for disease prevention and control to reduce morbidity, mortality and disability; provision of family health services to expand family planning, maternal, child and youth services; and promotion of environmental hygiene and sanitation. CHWs may be involved in a range of other tasks including home-based care, observed treatement and some curative tasks dependent on location (see Table 1). The CHW position is described as being linked to the primary health facility through the government employed community health extension worker (CHEW), a trained health worker employed in a link primary health care facility who provides support and supervision to 25 CHWs (2 CHEWs per unit of 50 CHWs and 5000 people) providing community health services (Figure 1 and Table 1). In addition a community health committee, consisting of voluntary community representatives, is described in the strategy. The committee conducts supervision and governance of CHWs and encourages community participation in health-related activities.

**Table 1:** CHW and CHEW details according to current and revised strategies

	Staffing per community unit	Selection and recruitment	Training	Tasks	Supervision
CHW current	50	Nominated by community but selection facilitated by community representatives. Must be able to read and write. Permanent resident within the community. Demonstrate attitudes valued by community.	Initial 10 day training followed by refreshers.	Community entry, organization, sensitization for 100 people Registering households, data gathering Collation of data on chalkboards Community dialogue for change Record keeping and report writing Health promotion Recognition and classification of common conditions and decision for action Home visiting Training and supporting home caregivers	Supervision by CHEW and community health committee.
CHW revised	10	Selected from pre-existing CHWs, community role in this unclear at present		Community mobilization.	Supervision by CHEW
CHEW current	2	Selected by government. Must have a health background such as nursing or public health.	5 days	Community entry, mobilization, organization and sensitization Establishing the information system, and the planning, implementation, monitoring, evaluation and feedback process Report writing Training of committees and CHWs Recognition and classification of common conditions and decision for action (treatment or referral) Home visiting communication through evidence-based dialogue Growth monitoring Supervision of 25 CHWs and supporting them in conducting the tasks described according to the needs of the community.	Officially supervised by multidisciplinary team including public health, public health nursing, environmental health and health education staff at district level.
CHEW revised	5	Proposed greater community role in selection, although how this will occur is unclear open to individuals with a basic certificate in social studies or community-related studies.	6 months Classroom and field training	Preventive, promotive, curative services.	Unclear supervisory structure.

Level 1 community services link directly with level 2 primary care services provided at health facilities (including dispensaries and health centres), through referral of patients by CTC providers from the community to the link primary care facility for a range of services from preventative (e.g. immunization and antenatal services) to curative (e.g. management of childhood illnesses). The 2010 Kenya Service Provision Assessment Report reveals that 73% of dispensaries and 81% of health centres provide all basic services. CHWs described that transferring patients to the health facility from the community was problematic (National Coordinating Agency for Population and Development *et al*. 2010) and attendance at peripheral health facilities in Kenya has been demonstrated to decrease with distance from a facility (Feikin *et al*. 2009).

Following introduction of the new constitution in 2010, policy formulation remains a function of the national government alongside developing standards and regulations. Meanwhile, public service provision (including health) was fully decentralized (devolved) from national level and is still being rolled out in the 47 county governments who currently have authority for decision making, adapting the policy to their local context, finance, implementation and management. The devolved health system is organized around a tiered system with community, primary care and county referral falling within the county’s responsibility (KPMG 2013). The community units remained linked to the larger health system as indicated in an organogram available on page 5 at http://www.who.int/pmnch/media/events/2013/kenya_hssp.pdf, which reveals the partnership, governance and stewardship relationships within the health system. When this study was carried out devolution was ongoing, with some responsibilities not yet handed over to county governments. The titles and positions of DHMT members had not been defined. These were later renamed into county and SCHMT members. The sub-county teams reported to the county teams while provincial health management teams were abolished. The county teams became the primary decision makers for programmes to be implemented in their counties, a shift from the previous model where the decisions were made at national level and implemented at provincial and district levels.

**Figure 2:**
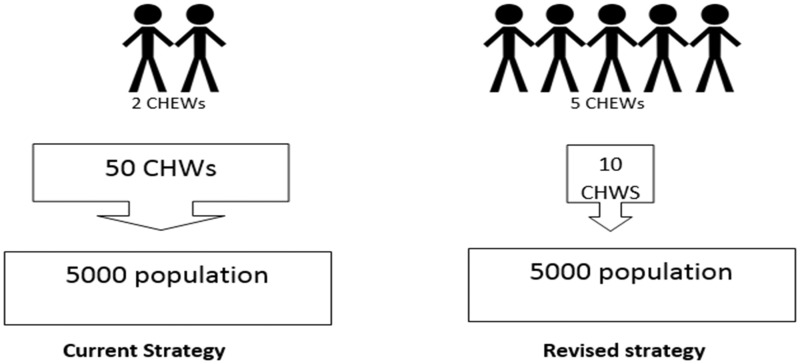
Current and revised CHS

The current community strategy is under revision. Despite recent evidence indicating improved behaviour change and utilization of health services in areas with community unit compared with areas without community unit (Olayo *et al*. 2014), there has been evidence indicating limitations with performance and constraints of the current system, such as high CHW attrition and conflict of workload for CHEWs (JICA *et al*. 2013) which provided the impetus for change. In the revised strategy, there will be five salaried CHEWs who will carry out promotive, preventive and curative tasks, supported by 10 volunteer CHWs (2 for each CHEW) who will now act as mobilizers, ensuring linkage between community and CHEWs for health-related activities for every community unit of 5000 people (Figure 2 and Table 1). This will result in an anticipated increase in the number of CHEWs nationally from 2100 to 25 000 by 2017.

The Five Wonders of Change Framework considers five elements of change (Why? What? Who? How? and What if?) providing a deeper understanding beyond typical linear models of change such as Beckhard and Harris’ model of change, which indicates a single transition state between the current state and the future desired state (Gittins and Standish 2010). We will adopt elements of the Five Wonders of Change Framework as a conceptual framework to inform the analysis and guide the interpretation and discussion of our findings.

While progress has been made developing a revised CHEW scheme of service and training curriculum, a number of unanswered questions remain. Unresolved queries include: the extent to which county governments, within the devolved system of service delivery, will adopt and budget for implementation of the revised strategy, including increased recurring CHEW salary costs; the definition of roles and responsibilities of both CHWs and CHEWs; the extent of curative or preventive services to be offered at each level and expectations of workload; quality assurance systems; supervision structures and the role of community in governance and accountability for CHEWs. As the revised strategy progresses towards implementation, a better understanding of the drivers for change, strengths and weaknesses of the current system and evidence-based recommendations are urgently required. The purpose of our research was to explore the drivers of policy change from the perspectives of policymakers and SCHMT members, as well as the perceptions of the current system from community to sub-county level.

## Methods

We used a descriptive exploratory qualitative design to generate rich data and explore CTC services from different perspectives within the context of policy revision in Kenya (Kuper *et al*. 2008). Two counties were selected, Nairobi and Kitui, to provide representation for both urban and rural contexts. The number and characteristics of study participants are shown in Table 2.

**Table 2:** Characteristics of study participants

Characteristics of health providers										
Type of provider			*Number of interviews*							
			*Total*			*Male*			*Female*	
Policymakers National			4			3			1	
DHMT members Nairobi			3			0			3	
DHMT members Kitui			3			3			0	
Facility in-charges Nairobi			2			0			2	
Facility in-charges Kitui			2			1			1	
CHEW Nairobi			8			4			4	
CHEW Kitui			8			4			4	
Characteristics of clients, community members and CHWs										

County	Number of Interviews	Female		Male	Education Level					
					None		Primary	Secondary		Tertiary

Kitui HBTC clients	5 IDIs	4		1	0		5	0		0
Nairobi HBTC clients	5 IDIs	5		0	1		1	2		1
Kitui community members	2FGDs	12		10	0		12	10		0
Nairobi community members	2FGDs	15		5	0		10	7		3
Kitui CHWs	3FGDs	25		11	0		19	15		2
Nairobi CHWs	3FGDs	24		12	0		10	18		8

Purposive sampling was used to select SCHMT members, facility managers and policymakers based on their knowledge and role in policy development and implementation of community health programmes. The community members and clients selection were based on residence in different community units where community health services are offered. CHWs and CHEWs were selected on the basis of being part of a community unit. To ensure diversity of respondents both female and male CHWs from a range of community units were selected to participate in each county. In-depth interviews were used with policymakers, SCHMT and CHEWs with the purpose of exploring strengths and weaknesses of the community services and barriers and facilitators to community provider performance with these respondents. Focus group discussions (FGDs) were used with CHWs and community members to use group interaction to aid in the generation of data (Pope and Mays 1995). Topic guides used with these groups are attached in Supplementary Appendix S1.

Data collectors received training in data collections and ethical considerations and the tools were translated into Kiswahili, back translated and piloted before use. To improve quality and trustworthiness of the data, a range of participants were interviewed and regular meetings were held between data collectors to discuss the data collection process and identify inconsistencies between findings of various participant groups. Preliminary findings were presented to the Ministry of Health and other stakeholders for community health services (such as NGOS and research institutions) to clarify and validate findings.

Written informed consent was obtained from all respondents before participating in the study. Data were digitally recorded and transcribed data were counterchecked with the audio files. A data analysis workshop involving all data collectors and other experienced qualitative researchers was held, where those involved in data collection assisted in the development and application of a common coding framework. The coding framework was based on a pre-existing framework developed during similar qualitative research conducted in Malawi. To ensure validity this was modified through an iterative process based on the new codes emerging from the Kenyan data and was also informed by the Five Wonders of Change Framework. All researchers who conducted coding were involved in adaptation of the framework to ensure common and shared understanding and application of codes. During the analysis process, regular debriefing sessions were held to discuss any further modifications. The framework method (Gale *et al*. 2013) was used to guide analysis and managed using the computer-assisted software Nvivo 10. Triangulation formed part of the final analysis, comparing findings between different respondent groups and sites to identify similarities and differences. The study protocol has received full ethical approval at all appropriate national and international institutions.

## Results

In general the CHS, and in particular the CHWs, were positively evaluated and appreciated by both health facility and community members, who reported adoption of healthy practices and improved relationships and linkages between the health facility and community level. Respondents at multiple levels discussed the commitment of community providers (both CHEW and CHW) to their role. Participants highlighted particular concerns and areas for improvement, reinforcing the drivers for strategy revision and raising additional unaddressed concerns. The results are structured against the key themes emerging from the analysis. The first section discusses perceptions of drivers of change and revised strategy details (key themes relating to this section include the need for change, plans to revise the CHS and uncertainty regarding change) and the second section presents perceptions of the existing strategy from community to sub-county level (including key themes relating to equity-related discussion and current strategy implementation).

### Drivers of change and revised strategy details from policymakers and SCHMT members' perspectives

The need for change, the plans to revise the CHS and uncertainty regarding how change will influence community health service provision were reflected in interviews with policymakers, SCHMT members, facility managers and in some instances CHEWs.

#### Need for change

Interviews with policymakers and SCHMT members focused around drivers for revision of the strategy, centring on current concerns about sustainability and workload, costs and weaknesses in the existing system and the desire for a more integrated and holistic approach. The current strategy is highly dependent on volunteer CHWs (there are only two paid government staff responsible for supporting and supervising 50 voluntary CHWs). As a result a number of policymakers and CHEWs considered that the absence of a CHW salary compromised sustainability of the strategy due to high rates of attrition among volunteer CHWs and an inability to hold CHWs accountable for their work:

*“Because CHEW’s are paid by the government you can hold them to account, rather than the volunteer who can leave an important job half way and you cannot hold him/her accountable because they were volunteering.”* (Policymaker 2).

Funding emerged as a sub-theme, widely raised by all types of participant and linked to weaknesses with the current CHS. The inability to pay all CHWs a salary was described as a driver for the restructuring within the revised strategy, which would result in increased numbers of salaried CHEWs and reduced numbers and responsibilities of CHWs to purely voluntary roles:

*“You see it will be costly … We have around 400,000 community health workers and you see this will not be financially viable. So what we are thinking of is how to restructure the community health work, so that the people that we should have working as community health workers are the community extension health workers, and have the community health workers purely as volunteers.”* (Policymaker 1).

The availability of funding from government was identified by a range of policymakers as critical for success, and this in turn was seen to depend on recognition of the effectiveness of the strategy on the part of treasury staff:

*“To me it is a good strategy that was meant to get health into the community however the government doesn’t fund the project hence [the strategy] hasn’t been successful. The treasury may not have been convinced that the project is worthwhile.”* (Policymaker 4).

#### Plans to revise the CHS and uncertainty regarding change

CHEWs were described as currently not playing an adequate role at community level, instead viewing themselves as supervisors only. In addition to the increased number of CHEWs, the change in CHEW tasks was also discussed by policymakers. The need for CHEWs to take on a more ‘hands on’ approach to the provision of services at community level was described:

*“They [CHEWs] are not seeing themselves as the community health providers, but they [CHEWs] are seeing the community health workers as the providers and they [CHEWs] as the supervisors and these are the things that we want changed.”* (Policymaker 1).

Reassuringly, almost all policymakers expressed a willingness to learn from weaknesses (to varying degrees) with the current strategy, which would be vital for success of the revised strategy:

*“I think that it is very important that the lessons and the challenges should inform the decision to revise the community strategy … so that we can come up with something that can work well for us and we can remove what we feel did not work well for us.”* (Policymaker 2).

### Perceptions of the existing strategy from community to sub-county level

There was limited discussion and awareness of the anticipated CHS revision at provider and community level. Providers and community members expressed appreciation for the existing CHS.

#### Equity-related discussion

However, while respondents from the community to policymaker level agreed that in general the introduction of the existing CHS has improved access to health services, there were still exceptions described, with some geographical areas in Kitui county not having a community unit established. Sub-county respondents described how implementing organizations selected which community units to work in, resulting in some of the most isolated units not receiving support. For example, one SCHMT member stated:

*“Respondent: There is poor distribution of the community units, you might find the district has got only one community unit, we have a district like Mumoni that does not have a community unit at all.*

Interviewer: And what is the problem, why is there poor distribution?

*Respondent: … through our partnership with our stakeholders, they facilitate the formation of the community units and majority of our stakeholders do not want to go far.”* (Kitui_SCHMT1).

Compounding geographical-access challenges, distance from the community to the closest government health facility, varied between areas. Some communities are located far from the link health facility and some informal settlement communities (in Nairobi) have no government facility available. This created a challenge for those referred by community providers to health facilities, particularly for those with limited funds for transport. Some respondents felt that the youth, men, people with disabilities or people who abuse drugs did not receive adequate services from the CHWs:

*“I know that there are some groups that they [CHWs] are not able to reach like the deaf, they don’t have the mechanism like how they are going to communicate with them, maybe the other group they are not able to reach are people who are abusing drugs, because this is a community that lives in a different world and they are feared in the community.”* (Kitui_SCHMT1).

There was a relatively widespread recognition of the benefit of integrated approaches for more holistic care among policymakers, SCHMT and CHEW respondents:

*“So that when we are attending to this client, we attend to all issues of nutrition, home based care issues, issues of tuberculosis, like that, so that when I come I come fully, not I come then another person comes for tuberculosis then another person comes, I just want to go and do everything.”* (Nairobi_CHEW8).

#### Current strategy implementation

Five sub-themes emerged relating to how the community strategy is currently implemented: community engagement, integration, supervision, incentives and workload.

#### Community engagement

One of the commonalities running throughout the data is the difference in depth of discussion by communities relating to CHWs and CHEWs. The vast majority of discussion focused around CHWs, with limited discussion by community respondents about CHEWs. While the whole community should be involved as far as possible in the selection of the CHWs, there is no role for community selection of CHEWs at present (although one policymaker did highlight the need for greater involvement of the community in future CHEW selection following strategy revision). At present CHEW selection is carried out by government or other health care workers, with this process being agreeable to most CHEWs.

The general perception of CHWs was positive, with community ownership and governance of CHWs facilitated through community health committees (CHC), where these were present and functioning. Meanwhile, there was no role described for the community to play in governance of the CHEW. In fact, one CHEW in Nairobi identified how he was rejected by the community who would not allow him to visit their homes because of a misconception that this was purely for his benefit, which could be a signal of the lack of community engagement in recruitment:

*“To your surprise you may find out that by the time you reach the household, the members are not there, because the public generally feel that any time a government representative visits, there is something benefiting this government official and not them, so they can resist loudly by saying “we are not giving you the information you want” or they can leave you there, that is usually in the urban set up.”* (Nairobi_CHEW3).

#### Integration of services

There were expectations expressed from all types of respondent demanding more integrated services at community level. A number of policymakers, SCHMT members and facility managers felt additional tasks could be shared at community level, but opinions ranged on who should conduct these—CHEWs or CHWs. Possible additional tasks described included malaria rapid testing and treatment, family planning, TB screening and home-based HIV testing and counselling (HBTC). In particular, HBTC was further probed to inform a subsequent study and findings relating to this will be presented elsewhere.

*“So that when we are attending to this client, we attend to all issues of nutrition, home based care issues, issues of tuberculosis, like that, so that when I come I come fully … .because these people in the community need care, they need people who can follow them up … so we need the integration.”* (Nairobi_CHEW8)

#### Supervision

Although supervision of CHWs and CHEWs is described in the strategy, the findings revealed it was often irregular and a diverse range of methods were used for CHW supervision: including monthly meetings, household visits and report review. The data indicated inconsistent use of the methods and poorly structured supervision. Some supervisors described challenges with lack of fuel and excessive workload which prevented them from carrying out aspects of supervision.

*“So far I have not held any house hold visits because, I don’t have that time since I work in the lab and most of the time I am in that facility. What I do is that I just supervise them from the lab. When they come I look at their work, whatever they have done, if it is not ok, I correct them I teach they on the approach they are supposed to use when holding a household visit, I have not done household visits so far.”* (Kitui_CHEW1)

Further, there was no discussion as to how CHEWs themselves were supervised, with the exception of one policymaker who admitted this had not been planned. Many CHEWs discussed how they would like to receive supervision to further develop their skills:

*“I think it [supervision] should be from the higher level downwards because we also want to learn something, so I think one of the coordinator should come and do the supervision with us.”* (Kitui_CHEW1).

#### Incentives

Non-financial incentives play a key motivating role for both CHWs and CHEWs who participated in this research, a fact which has been exploited through necessity by the current strategy, with CHWs drawing on a sense of pride from being a role model, achievement from seeing community behaviours change, recognition from supervisors, community and peer support rather than financial rewards as their source of motivation:

*“I feel so good because I can see my progress. I can evaluate my performance based on the positive changes in the community.”* (Kitui_CHEW1).

In fact, some providers (both CHWs and CHEWs) are so motivated by these non-financial incentives that they paid out of their own pockets to help community members receive the health care they need:

*“… in our location, there is no health centre. The hospitals that are present are private and as I earlier told you we as CHWs contribute to pay the medical bills of our community member …”* (Nairobi_CHW1).

The absence of a salary was viewed as a de-motivator by most CHWs and the need for alternative funding sources was discussed, with income generating activities being commonly described by CHWs across both counties. Almost all CHEWs and SCHMT felt that the CHEW salary was inadequate. The CHEW salary is US$ 450–500 per month, similar to other health workers of a similar cadre, all of whom feel the salary is inadequate. One CHEW describing how this made it difficult to perform well:

*“The amount that I am receiving cannot sustain me because you can only perform well if you are comfortable, for you to be comfortable you have to have all the basic needs and everything goes with money.”* (Nairobi_CHEW3).

#### Workload

There was a general consensus that workload for both CHWs and CHEWs is too high. For CHWs this was attributed to their voluntary status, with the lack of earnings for CHWs creating challenges:

*“You find that the CHW has a lot of work to do and most of the times they can forget their households we work 24/7. … others are single mothers with no earnings and the married ones compromise their family time as they have a lot to do in the community.*”(Naibobi_CHW2)

Meanwhile, for CHEWs there was conflict of role described, with many CHEWs, particularly in Kitui county, having a role at the health facility as well as in the community, which created lack of clarity for some CHEWs regarding how to balance their workload.

*“… CHEWs find a lot of challenges because they are now torn into two. They attend to the community and to the facility as well”* (Kitui_CHEW4).

## Discussion

Our findings capture multiple perspectives on the current CHS in Kenya and result in robust recommendations for strategy revision. The study design allowed for exploration of community and provider voices as well as the more established views of supervisors and policymakers. Their combined recommendations point towards a more people-centred health system for improved equity and effectiveness and indicate priority areas for action if success of policy change through the roll-out of the proposed revised strategy is to be realized. The proposed policy change, grounded in the recognition of the limitations of the current system, is already seeking to address key challenges, relating to financial incentives and workload, through the very nature of the revision itself with increased number and community role for CHEWs. However, incentives, workload, equity, community engagement with local governance of CHEWs, integration and supervision, will need renewed emphasis for success of the strategy revision.

The critical reflection and honesty shown by policymakers in acknowledging funding gaps is crucial to advocate for future, comprehensive funds for community health services. A recent cost-effectiveness study conducted in Kenya, Indonesia and Ethiopia indicated that CHW programmes in contexts where they work with an integrated team supported by the health system have a high likelihood of being cost-effective (Edoka *et al*. 2014; Global Health Workforce Alliance 2014). Following strategy revision, counties will need to budget for greater recurring costs due to the increased numbers of salaried CHEWs. In keeping with a study of the Kenyan CHS by Japan international cooperation agency (JICA) (2013), our findings revealed that providers (CHEW and CHW) expect some form of financial incentive for their work and that provision (or lack) of incentives influenced community provider performance, attrition and accountability. Glenton *et al*. (2010) found that incentives need to be context-specific and aligned to providers’, managers and policy managers’ expectations if they are to tackle attrition and performance. It is therefore reassuring that respondents were in agreement regarding the need for financial incentives. The coalition government indicated in their pre-election manifesto their support for preventive services and community health and expressed commitment to increase health spending from 6 to 15%, (The National Alliance *et al*. 2013) However, this needs to translate to action as for 2013–14 financial year the Government of Kenya had not budgeted to cover CHEWs’ salaries. Workload expectations for CHWs and CHEWs were often identified as high and conflicting for CHEWs. High workload is in keeping with findings from a recent systematic review (Glenton *et al*. 2013). Through the strategy revision process the issue of dual workload for CHEWs should be eliminated. However, it will be important that workload is monitored given the increased community responsibilities for CHEWs.

To ensure the revised strategy meets targets of increasing equitable coverage as specified in the Kenya Health Sector Strategic Plan (Republic of Kenya 2013), strategies to reach more vulnerable groups need to be included in the training and supervision of community providers, with equity indicators (including gender, age, geography, dis/ability and where possible proxies for poverty) included within health information systems, to capture the impact of the revised strategy to ensure that attention remains focused on equity concerns (Theobald *et al*. 2009).

Implications for the process of policy change management, transitioning from the current to the revised strategy need specific consideration. Guided by the Five Wonders of Change Framework, the results of this article have outlined the WHY of change by exploring the perceptions of drivers for change and the WHAT of change, by describing the revised CHS, how this was discussed by policymakers and what the perceived vision for change is. The following section outlines the three remaining aspects (who, how and ‘what if’) of change management in further detail (Gittins and Standish 2010):

The WHO of change—the success of making difficult changes is dependent on the effective involvement of key groups and individuals, however—awareness of the strategy revision was low during discussions at community level. The community, which have only recently embraced the existing strategy, will now face further changes. This is especially significant as the revised strategy plans to reduce the number and role of CHWs whom the community selected, and the perceptions of the community for the changing role of CHWs (who are valued by communities) will be crucial for sustainability of the revised strategy. Several studies have highlighted that selection of providers by their community is felt to have contributed to the success of community programmes (Elmardi *et al*. 2009; Brenner *et al*. 2011) and community support, feedback and social prestige as a result of community work are described in international literature as important non-financial motivating factors for community workers (Osawa *et al*. 2010; Alam *et al*. 2012). Kenya already has a well-established community engagement system described within the existing strategy (Ministry of Health 2006), which was valued by respondents through our research. This provides a suitable structure to use for introducing and promoting acceptance of the revised strategy with the greater community role for CHEWs and lesser role for CHWs.

The HOW of change—in order for change to be successful there needs to be good process thinking and planning for each phase. The integration of tasks for more holistic care by community providers was identified as a priority for policymakers during this research. One task which dominated discussions, particularly at community and provider level, is the integration of HBTC. Often disease-specific programmes are conducted vertically, often by NGOs, under supervision by government staff. Strategy revision presents a unique opportunity to pilot integration of traditionally vertical programmes within existing national structures. It will be vital that there is an emphasis on quality throughout any future integration.

The presence of effective supervision has been described in international literature as influencing the quality of community service provision, providing opportunity for motivating, giving feedback and guiding community providers (Heaman *et al*. 2006; Nankunda *et al*. 2006; George *et al*. 2009; Daniels *et al*. 2010; Dynes *et al*. 2011). While the Kenya CHS (2006) describes use of a multidisciplinary supervision team including regular performance appraisals based on checklists to measure performance, there are no guidelines provided on frequency, supervision avenues or how to use data collected, resulting in limited use of data during supervision. By comparison, a clear and consistent supervision structure with a range of tools have been developed by government in collaboration with stakeholders, based on national quality management guidance and used at regular intervals for supervision of home-based HIV Testing and Counselling (HTC) counsellors (LVCT Health 2013). Home Based Testing and Counselling (HBTC) is presently conducted as a vertical programme, with supervision carried out by Non Governmental Organisation (NGO) staff (although HBTC activities are supervised within each sub-county by district AIDS and Sexually Transmitted Infection (STI) coordinator who is a government employee). 

The WHAT IF of change—deliberately thinking through and identifying potential risks in advance can identify potential solutions and thereby prevent or minimize risks. Devolution brings with it unique opportunities and challenges for each county to influence equity of health service provision, since decision making is brought closer to communities. Opportunities include the ability to prioritize services within the county for more equitable coverage, addressing county specific health burdens, potential greater coordination between actors and opportunity for stronger community participation at county level. Indeed, this need for county-specific approaches has recently been highlighted by Ochieng *et al*. (2014) who identified the need for task-shifting, training and motivational strategies for CHWs to be context-specific given differences noted between rural, nomadic and peri-urban settings in Kenya (Ochieng *et al*. 2014). However, devolved county authorities also have authority to prioritize services and resources which may result in greater rather than reduced inequities (KPMG 2013). Potential additional challenges associated with devolution include delays in revised policy implementation, challenges to implementation as a consequence of conflict between national and county governments and possible resistance to the revised strategy due to salary costs associated with the greater numbers of CHEWs, who are now the responsibility of the county governments. With coordination and management of the delivery of health services at county level there is therefore need for excellent communication and advocacy at county government level to demonstrate benefits of the revised strategy and financial investment in community health to ensure adequate budgeting for the provision of recurring costs needed to fund implementation of the revised strategy. This research identified poor coverage of community units in Kitui, creating challenges in ensuring equitable service coverage. Before devolution, there was poor coordination because of the double role of national and county governments. Devolution provides the opportunity for better coordination of county level service delivery among stakeholders as they must report and get approval from the counties. This is however dependent on the capacity of the counties, since there is the potential for poor coordination between stakeholders resulting in parallel structures, gaps and overlaps, particularly of vertical programmes (KPMG 2013).

This study has generated rich data from a range of respondents that provides unique and timely insights into perspectives of ongoing CHS revision. However, our study had a number of limitations. Topic guides were translated into Kiswahili; however, in Kitui county, some community respondents struggled to communicate in Kiswahili, which may have affected their ability to effectively provide the needed information. The CHWs and community groups were identified by CHEWs who may have selected active CHWs or those with whom they have good relationships. In general, it was difficult to find enough male CHWs and clients for the FGDs. FGDs were held with active CHWs only. It would have been interesting to know more from those CHWs who have quit to better understand their reasons for doing so. Devolution is not thought to have significantly influenced the responses from the SCHMT in this study as there was no major restructuring at sub-county level. In addition, we did not ask specific questions relating to devolution, which is an influential factor for strategy revision.

## Conclusion and Recommendations

**Figure 3.**
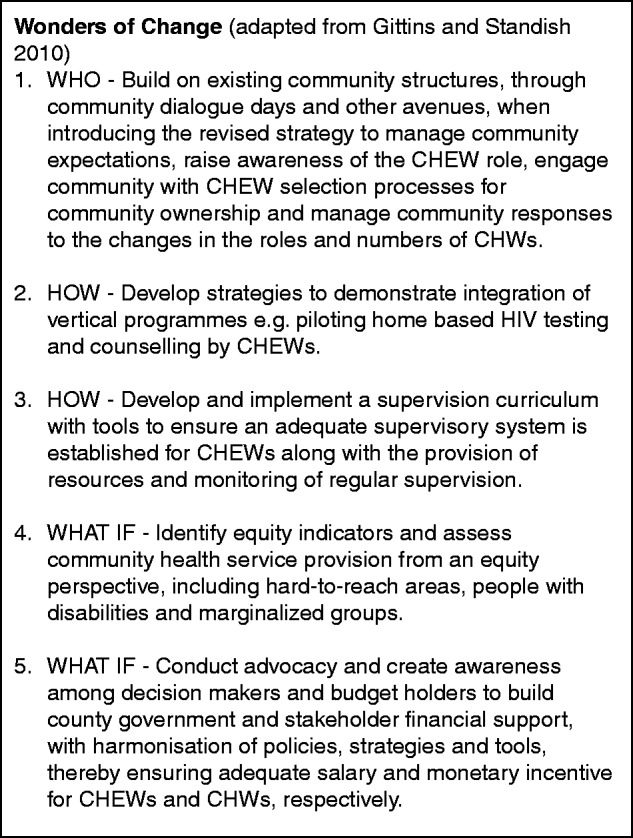
Recommendations for strategy revision based on WHO, HOW and WHAT IF of Five Wonders of Change

Our study reviewed a wide range of perceptions of the Kenyan CHS from the community to the national level and identified critical factors for policy revision. Some of these factors have already been addressed and some remain to be addressed. In particular the need for equitable, integrated services; the sustainability of a predominantly voluntary workforce and financing challenges associated with providing a salary for all CTC providers under the existing strategy were highlighted. We identified future implications for the process of change management including, management of provider expectations and decision makers' support at county level, community and stakeholder engagement with strategy revision, integration of services and equitable roll-out in light of devolution. Five recommendations shown in Figure 3 are proposed before roll-out of the revised strategy.

## Ethical Approval

The study protocol was approved by both the Kenya Medical Research Institute Ethics and Review Committee (non-SSC protocol number 399) and the Royal Tropical Institute (KIT) Research Ethics Committee (approval number REC KIT S45).

## Supplementary Data

Supplementary data are available at *HEAPOL* online.

Supplementary Data

## Notes

^1^ A CTC) provider is a health worker who carried out promotional, preventive and/or curative health services and who is the first point of contact at community level. The provider can be based in the community or in a basic primary facility. A CTC provider has at least a minimum level of training in the context of the intervention that they carry out and not more than 2–3 years para-professional training.
