# Supplementary material for: Exploring perceptions of community health policy in Kenya and identifying implications for policy change
Source: Health Policy Plan. 2015 Mar 26;31(1):10–20. doi: 10.1093/heapol/czv007 (PMC4724165; doi:10.1093/heapol/czv007)
Supplement: Supplementary Data [file supp_31_1_10__index.html]

Exploring perceptions of community health policy in Kenya and identifying implications for policy change — Exploring perceptions of community health policy in Kenya and identifying implications for policy change — Supplementary Data 

# Exploring perceptions of community health policy in Kenya and identifying implications for policy change

## Supplementary Data

files

**Files in this Data Supplement:**

- Supplementary Data - docx file
