# Supplementary material for: Exploring perceptions of community health policy in Kenya and identifying implications for policy change
Source: Health Policy Plan. 2015 Mar 26;31(1):10–20. doi: 10.1093/heapol/czv007 (PMC4724165; doi:10.1093/heapol/czv007)
Supplement: Supplementary Data [file supp_czv007_Appendix_1.docx]

**Appendix 1: Topic Guides**

FOCUS GROUP GUIDE FOR COMMUNITY MEMBERS (ENGLISH)

**Introduction**

1. Are you aware of any community health workers (CHW) providing services in your village?

2. What services do they provide?

3. Are there types of CHWs who only offer specific services in your community?

(*probe for types of CHWs)*

4. Do CHWs visit everyone in your community or just some homes and why?

5. Do you feel CHWs should visit everyone in the community?

6. How often do CHWs visit homes in your community?

**Recruitment**

7. Do you know how the CHWs were selected?

8. What makes a good CHW?

9. Were you involved in the selection of the CHWs?

**Tasks**

10. What do you think are the functions of a CHW?

11. Are there tasks they do that you think they should not be doing? Are there tasks they do not do that you think they should be doing?

12. If you were given the chance, would you want to be a CHW? Why? Why not?

**HIV Services**

13. What HIV services are provided in this district? Which of these services is carried out in the community?

*(probe on VCT, Couple Counselling and Testing, HIV Care and Treatment)*

14. Have you heard about home-based testing and counselling (HBTC)?

15. Do you think that CHWs should offer HBTC?

**Quality of care**

16. How is follow-up on services provided by CHWs done, how is their work supervised?

17. What is good about the services that CHWs offer in your village?

18. What changes would you like to see effected in the services that CHWs offer in your village?

19. Do you think that CHWs keep the information that they collect confidential? Would you be comfortable sharing personal health issues with a CHW?

20. Other than visiting households, what other interactions do you have with the CHWs?

21. Do you think that CHWs should offer more services?

FOCUS GROUP GUIDE FOR COMMUNITY HEALTH WORKERS (ENGLISH)

**Introduction**

1. Please tell us what you do as a community health worker?

2. What are your key tasks during the week?

3. How much time do you spend each week carrying out your duties?

*(Probe for whether there is an overspill of CHW work beyond the designated hours they are recommended to carry out their duties).*

4. What do you spend most of your time on?

5. How do you feel about the amount of time you spend on carrying out your duties?

**Recruitment**

6. How were you recruited as CHW? What criteria were used for selection for your job?

7. How did you find out about the job of a CHW?

8. Did you receive formal training for your role as a CHW? Who carried out the training? How long did the training take?

9. How useful was the training in relation to the work you do?

*(probe for what has been put to use from training and what has not been useful)*

10. Which activities are you involved in that were not covered in training or require further training?

**Motivation**

11. What do you like about being a CHW?

12. What don’t you like about being a CHW?

13. Why did you decide to take this job?

14. What is it about this job that encourages you to continue working as a CHW?

15. What would discourage you from continuing your job as CHW?

**Supervision**

16. Who do you report to?

*17.* How do they supervise your activities?

*(Probe for whether there are any visits to households with supervisor, supervision meetings, etc.)*

18. How frequently do you meet your supervisor?

19. Who do you go to when you have a problem in carrying out your duties?

*(Probe for whether it is someone other than the supervisor)*

20. What do you think about the supervision that you receive? How can this supervision be improved?

**Tasks**

21. What are the functions of a CHW?

*(Use checklist of tasks)*

22. Are there tasks that you are asked that you should not be doing? Are there tasks that you are not doing that you think you should be doing?

23. Do you think that CHWs can take on more duties?

*(If yes, probe for examples of what these duties could be)*

SEMI STRUCTURED INTERVIEW GUIDE FOR CLIENTS (ENGLISH)

**Introduction**

1. Do community health workers (CHW) provide services in your village?

2. What services do they provide?

3. How often do CHWs visit your homes?

**Tasks**

4. What do you think are the functions of a CHW?

5. Are there tasks they do that think they should not be doing? Are there tasks they do not do that you think they should be doing?

**HIV Services**

6. What HIV services are provided in this district? Are any of these services is carried out in the community?

7. Have you heard about home-based testing and counselling (HBTC)?

**For those who have had HBTC in the past:**

8. When did you have HBTC?

9. Can you tell me about the experience?

*(Probe for positive and negative aspects of the experience)*

10. Did you get your results immediately?

*(****Do not*** *ask for result)*

11. What happened after you received your results?

12. Would you recommend HBTC to someone else? Why?

**For those who have NOT had HBTC in the past:**

13. Would you be interested in HBTC being offered in your village?

14. Have you ever been offered HBTC?

15. *(If yes)* Why did you refuse?

**Quality of care**

16. What is good about the services that CHWs offer in your village?

17. What is bad about the services that CHWs offer in your village?

18. Do you think that CHWs keep the information that they collect confidential? Would you be comfortable sharing personal health issues with a CHW?

19. Other than visiting households, what other interactions do you have with the CHWs?

20. Do you think that CHWs should offer more services?

SEMI STRUCTURED INTERVIEW GUIDE FOR COMMUNITY HEALTH EXTENSION WORKERS (ENGLISH)

**Recruitment of formally engaged CHEWs**

1. How were you recruited as CHEW?

(*Probe for: role community, health professionals, ministry, NGO/CBO)*

2. What criteria were used for selection for your job/role? Do you think this was the correct criteria for CHEW selection?

3. If you had to make the criteria for new providers what changes would you make, if any, in the criteria or process of recruitment?

(*Probe for: skills and community involvement)*

**Incentives and motivation**

4. What things make makes you feel good or not so good about your work?

5. What things influence your job satisfaction as a CHEW?

(*Probe for: workload, working environment, communication, colleagues, and other health* **extension** *workers)*

**Tasks**

6. What things influence how the CHWs feel about the tasks they carry out?

(*Probe for: expectations of community and CHWs, supervision, what happens if something goes wrong?)*

7. How do you supervise CHWs?

(*Probe for: What do you like about supervision and what do dislike about supervision? How often are they supervised? When was the last time? What happened?)*

8. How are you enabled and limited in your supervision of CHWs at work?

*(Probe for: Influencing decision making, feeling powerless, problem solving process)*

**Quality of care**

9. What do you think about the quality of services provided by CHWs?

10. How is the quality of their work evaluated? By who? How?

*(Probe for: guidelines, protocols, monitoring of quality)*

11. What do you think people in the community think about the quality of services CHWs provide? Would they want CHWs to provide more services?

**HIV services**

12. What HIV services do the CHWs in your community unit provide?

*(Probe for: referrals and linkages, defaulter tracing)*

13. What do you know about home based HIV testing and counselling (HBTC)?

14. Do you think that it is a service that should be integrated into the community strategy?

15. Do you think that CHWs should be trained to offer HBTC? Why?

*(Probe for: skill set, training, quality assurance)*

**Facilitators and barriers**

16. What do you think goes really well in your work as a CHEW?

*(Probe for examples)*

17. What do you think does not always go well?

*(Probe for: CHEW and CHW workload, supervision structure, data use)*

**Lessons learned, opportunities constraints**

18. What can be done to improve your work as a CHEW?

**M&E**

19. What records do you keep of your work?

20. How is this information collected?

21. What do you do with this information? What happens with this information?

22. Do you get feedback about the results of your work? If so, how is this communicated and by whom?

**HIV Knowledge**

24. What HIV services are provided in this district? Which of these services is carried out in the community?

25. Do you offer any HIV services as part of your work as a CHW? Which ones?

26. Have you heard about home-based testing and counselling (HBTC)? If yes, what does it consist of? Have you been involved in HBTC? In what capacity?

27. Do you think that you can offer HBTC as a CHW? What skills would you need to learn in order to offer HBTC?

**Quality of care**

28. What do you consider to be quality service? What do you think about the quality of the services you give the community?

29. How does your CHEW insure that you are providing quality services?

30. What do you think people in the community think about the service that you provide? Do you think that they want you to provide more services?

31. Other than visiting households, what other interactions do you have with the community in your role as a CHW?

32. What do you do when you have a problem in your work?

**Facilitators and barriers**

33. What do you think goes really well in your work as a CHW?

34. What do you think does not always go well?

35. What can be done to improve your work as a CHW?

36. What do you require to help you do your job?

**Remuneration**

37. What do you think about your remuneration?

**M&E**

38. What records do you keep of your work? How is this information collected?

39. What do you do with this information? Do you get feedback about the results of your work? If so, how is this communicated and by whom?

40. Who do you report your activities to?

SEMI STRUCTURED INTERVIEW GUIDE FOR POLICYPOLICY MAKERS AND DHMT MEMBERS

**Knowledge of CTC programmes**

1. What do you know about the community strategy?

*(Probe for: guidelines and policies)*

2. Which community based providers are you aware of?

*(Probe for: mentor mothers, peer educators etc.)*

3. What guidelines for CHW and CHEWs are you aware of? In your opinion, what are the most important aspects of these guidelines? What could be improved?

4. What do you know about HIV Testing and Counselling services and Home Based Testing and Counselling services?

5. Is it possible to integrate HIV services into the community health strategy? How do you think this should be done?

6. What is the level of integration of HIV services in the community strategy?

*(Probe for: HIV training for CHWs, referral and linkages, defaulter tracing, mobilisation, health education, other players)*

**For DHMT members only:**

7. Are you in direct contact with CHW/CHEWs?

8. What is your role?

*(Probe for: tasks, how often in contact, for what)*

9. What is the role of the DHMT in ensuring delivery of the community strategy?

(*Probe for: motivation, training, supervision, remuneration)*

**Facilitators and barriers**

10. What do you think is the importance of the community health strategy?

11. Do you see any weaknesses in the community health strategy?

12. What do you think goes really well in the community health strategy?

*(Probe for: a pathway, flow of events.)*

13. How do you ensure that standards are being maintained in community based services?

*(Probe for: supervision, quality assurance mechanisms)*

**Lessons learned, opportunities constraints**

14. What are the challenges faced in implementing the community health strategy?

15. What can be done to address these challenges?

*(Probe for: HRH and QA and M&E)*

**HRM**

16. How are CHWs and CHEWs recruited and what are the criteria for selection?

17. What do you think about the tasks of CHWs and CHEWs?

18. What is your opinion on their workload? What tasks should be retained and which ones should be changed?

19. Tell me about their:

- Incentives
- Remuneration
- career perspectives
- training and continuing education
- supervision

**Motivation**

*20.* What factors influence job satisfaction and motivation of CHW and CHEWs?

*(Probe for: equipment and supplies, workload, working environment, communication, equipment and transportation, safety and sexual harassment, career perspective, supervision, community, clients, colleagues, other health workers)*

**For DHMT members only:**

21. What factors influence the perceptions of the providers on their tasks?

*(Probe for: expectations of community, clients, other providers and supervisors; how they feel about meeting these expectations, worries, concerns, what happens if something goes wrong, if a client complains?)*

22. Who are the clients of the CHWs? Is any group left out?

23. Are there issues around stigmatisation of clients, kindly explain?

**Referral**

24. How is referral organized in the community health strategy?

*(Probe for: different referral processes for different condition, ask for examples.)*

25. What goes well and not so well in referral?

**M&E**

26. How is the information about performance of community health strategy programmes collected?

27. What communication channels are used?

28. What happens with this information? Do you give feedback about the results of the work?
